# Development of synthetic lethality in cancer: molecular and cellular classification

**DOI:** 10.1038/s41392-020-00358-6

**Published:** 2020-10-19

**Authors:** Shijie Li, Win Topatana, Sarun Juengpanich, Jiasheng Cao, Jiahao Hu, Bin Zhang, Diana Ma, Xiujun Cai, Mingyu Chen

**Affiliations:** 1 Department of General Surgery, Sir Run-Run Shaw Hospital, Zhejiang University School of Medicine, 310016 Hangzhou, China; 2 School of Medicine, Zhejiang University, 310058 Hangzhou, China; 3 Key Laboratory of Laparoscopic Technology of Zhejiang Province, 310016 Hangzhou, China; 4 Zhejiang Minimal Invasive Diagnosis and Treatment Technology Research Center of Severe Hepatobiliary Disease, 310016 Hangzhou, China; 5 Zhejiang Research and Development Engineering Laboratory of Minimally Invasive Technology and Equipment, 310016 Hangzhou, China; 6 Zhejiang University Cancer Center, 310000 Hangzhou, China

**Keywords:** Cancer therapy, Cancer genetics, Oncogenes, Cancer metabolism

## Abstract

Recently, genetically targeted cancer therapies have been a topic of great interest. Synthetic lethality provides a new approach for the treatment of mutated genes that were previously considered unable to be targeted in traditional genotype-targeted treatments. The increasing researches and applications in the clinical setting made synthetic lethality a promising anticancer treatment option. However, the current understandings on different conditions of synthetic lethality have not been systematically assessed and the application of synthetic lethality in clinical practice still faces many challenges. Here, we propose a novel and systematic classification of synthetic lethality divided into gene level, pathway level, organelle level, and conditional synthetic lethality, according to the degree of specificity into its biological mechanism. Multiple preclinical findings of synthetic lethality in recent years will be reviewed and classified under these different categories. Moreover, synthetic lethality targeted drugs in clinical practice will be briefly discussed. Finally, we will explore the essential implications of this classification as well as its prospects in eliminating existing challenges and the future directions of synthetic lethality.

## Introduction

**Fig. 1:**
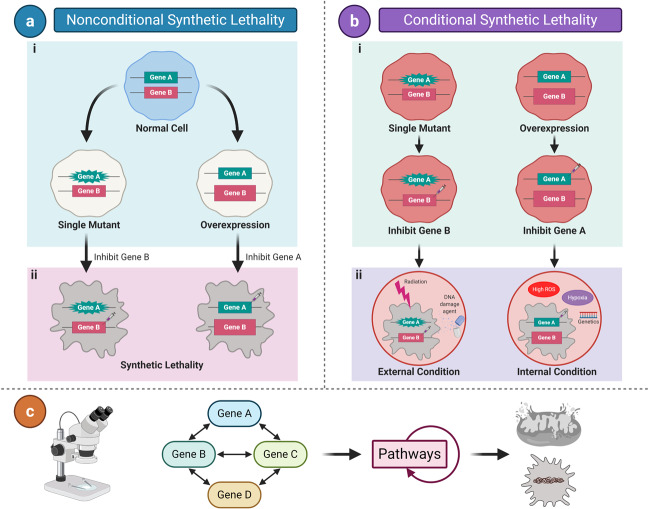
Synthetic lethality classification. Synthetic lethality is divided into two major categories, nonconditional synthetic lethality and conditional synthetic lethality. **a** Nonconditional synthetic lethality. (i) Single mutation/overexpression of either gene A or B alone is viable in tumor cells. (ii) Inhibition of gene B or A in cells with a mutation/overexpression of gene A or B results in synthetic lethality. **b** Conditional synthetic lethality. (ii) Several synthetic lethal interactions may be dependent on certain intrinsic conditions, such as genetic background, hypoxia, high ROS, etc., or extrinsic conditions, such as DNA-damaging agents and radiation. (i) Without these conditions, tumor cells with mutation/overexpression of both gene A and B could still survive. [c] Nonconditional synthetic lethality was further classified into gene level, pathway level, and organelle level according to the degree of studies into its mechanism in the review. Star shape of genes represents mutations; large rectangle represents genetic overexpression; syringe represents inhibitors; viable cells are depicted as ovals; and non-viable cells are depicted in random shapes

Synthetic lethality (SL) initially originates from studies on fruit flies^1,2^ and yeast^3–5^ models. The original concept of SL is based on the simultaneous occurrence of abnormalities in the expression of two or more separate genes, including mutation, overexpression, or gene inhibition, which leads to cell death; whereas abnormality in only one of the genes does not affect cell viability (Fig. 1a).^6–8^ Tumor cells are the result of mutated or overexpressed genes in otherwise normal cells.^9^ Hence, inhibitors that target synthetic lethal partners of mutated or overexpressed genes in tumor cells can kill cancers without affecting the survival of normal cells.

With the advancement of tumor research, cancer is now widely recognized as a disease of the genome. Various underlying tumor features, such as genome instability, give rise to the genetic diversity that accelerates their acquisition and inflammation.^10^ Therefore, targeting oncogenic driving genes, tumor-suppressor genes, and the underlying mechanisms is an applicable direction for cancer therapy.^11^ The development of genome sequencing and the analysis of thousands of human tumors led to the discovery of the first generation of genetically targeted cancer therapies.^12–14^ As a result, multiple personalized or precise genotype-targeted cancer treatments have been adopted and shown promising results in cancer patients that failed to respond to standard therapies.^7,15,16^ For instance, several studies have demonstrated that imatinib, a KIT inhibitor that is effective in treating patients with KIT-mutant gastrointestinal stromal tumors, had approximately 50% response rates and an extended median progression-free survival of 1.5 years.^17–20^ Imatinib also targets the BCR-ABL fusion tyrosine kinase for patients with chronic myelogenous leukemia.^21–24^ There are multiple studies that exhibit successful clinical outcomes,^11^ such as trastuzumab that target encoding HER2 in breast cancer,^25^ erlotinib, or osimertinib for EGFR mutations in non-small-cell lung cancer (NSCLC), as well as crizotinib for ALK-positive lung cancer, and others.^26–30^

Although numerous small-molecule and antibody-based drugs for oncogenes or tumor-suppressor genes have proven to be effective for several tumors with certain gene mutations,^31^ not all oncogenes or tumor-suppressor genes could be targeted and resistance is common,^7^ In such cases, identifying and exploiting a second or several other functional genes that interact with the primary oncogene or tumor-suppressor gene provides an alternative method for cancer treatment. Therefore, SL is increasingly being explored recently, in an effort to identify new anticancer therapeutic targets through large-scale SL screening in model organisms and human cell lines such as NSCLC (NCI-H1355, NCI-H1299, NCI-H1155), hepatocellular carcinoma (HCC1954, HCC1937, HCC1806), and breast cancer (MDA-MB-468, MDA-MB-436, MDA-MB-415) via clustered regularly interspaced short palindromic repeats (CRISPR),^32^ tumor genomic sequence database, RNA interference (RNAi) technology,^33,34^ etc. The most remarkable finding in SL is the hypersensitivity of BRCA1/2-mutant tumor cells to poly-(ADP-ribose) polymerase (PARP) inhibitors.^35–37^ Several PARP inhibitors (PARPi) were approved by the FDA for the treatment of breast cancer and ovarian cancer in clinical practice.^6,38^ Furthermore, there have been various findings regarding classical oncogenic driving genes or tumor-suppressor genes, such as TP53, KRAS, MYC, etc.,^39^ which will be discussed in detail later.

As our understanding of the complexity of cancer-cell signaling networks continues to grow, increasing numbers of targets are being identified as potential synthetically lethal candidates. Many researchers have defined several classes of SL into “synthetic dosage lethality (SDL)”,^7,40,41^ “collateral SL”,^42,43^ and others due to its complexity, which is different from the original concept of SL. However, these expanding concepts are scattered and there is no comprehensive classification for various SL that have been discovered. Moreover, the application of SL in clinical practice still faces many challenges. This review will describe a novel and integrated classification of the SL in different situations for a deeper systematic understanding. Multiple SL studies in recent years will be reviewed in different synthetic lethal categories. Furthermore, SL targeted drugs in clinical practice will be briefly discussed. Lastly, the development and limitations of SL as well as the inspiration of this classification for advancements in cancer research will be summarized.

## Synthetic lethality classification

This novel classification categorizes SL into various groups that comprehensively summarize different conditions involved in SL reported in recent years that are beyond the original concept. SL is generally divided into two major categories, nonconditional/original SL and conditional SL (Fig. 1a, b). Nonconditional SL is further classified into gene level, functional pathway level, and organelle level, according to the degree of specificity into its biological mechanism (Fig. 1c), while numerous achievements of SL in recent years are reviewed under different synthetic lethal categories we propose accordingly. The existing concepts of SL, namely synthetic dosage lethality, collateral SL, and metabolic SL, are discussed and grouped into this novel classification correspondingly.

## Synthetic lethality in genetics

Gene level SL corresponds with the original concept of SL mentioned above (Fig. 1a), in which the interaction between genes forms the basis for SL. The identification of genes with synthetic lethal effects allows researchers to further study the mechanisms at deeper levels. Thus, this category encompasses most of the studies on SL and is the cornerstone of SL at the other levels we mention below. Herein, we focus on describing the examples of oncogenes and tumor-suppressor genes related to SL at the gene level. The significant preclinical findings of synthetic lethal interactions among genes are listed in detail in Table 1.

**Table 1:** Representative synthetic lethal interactions among genes in preclinical studies

Gene	Chromosome	Cellular process and mechanism	SL partners	Cancer type	Reference
PARP1 (mutant)	1q41.42	Regulate cell proliferation and differentiation; repair DNA single- and double-strand breaks.	BRCA1/2	Breast, ovarian, pancreatic and liver cancer; leukemia	^6,50,132,138^
			RAD51	Ovarian cancer; HCC	^139,140^
			ATG5	Ovarian cancer	^141^
			CDK5	Cervical and breast cancer	^142,143^
TP53 (mutant)	17p13.1	Major tumor suppressor; regulate the cell cycle, senescence, and apoptosis.	ATM	Glioma	^54^
			ATR	CLL; osteosarcoma, colon and breast cancer	^55,56^
			WEE1	HNSCC	^57^
			CHK1	NSCLC, B-ALL	^58,144^
			BCL-2	AML	^59^
			SLC711	NSCLC; renal, esophagus, cervical and gastric cancer	^145^
			mTOR	Pancreatic adenocarcinoma; lung and breast cancer	^102^
			AURKA	Liver cancer	^146^
			PIP4KB	Breast Cancer	^147^
KRAS (mutant)	12p12.1	Transcriptional activator that regulates endothelial cells endothelin-1 gene expression.	CDC6	Colon cancer	^63^
			GATA2	Colon cancer; NSCLC	^63,64^
			SLC25A22	Colorectal cancer	^65^
			PLK1 and ROCK	Lung and pancreatic cancer	^66^
			CD274	Colon and lung cancer	^67^
MYC (mutant)	8q24.21	Regulate cell cycle progression, transcription, and apoptosis.	4EBP1	Hematological cancer	^68^
			SAE1/2	Breast cancer	^69^
			AURKB	T-ALL	^70^
			PIM1	Breast cancer	^71^
			CDK9	HCC	^72^
ARID1A (mutant)	1p36.11	Target SWI/SNF complexes, which regulate chromatin remodeling. SWI/SNF complexes are involved in controlling the cell cycle, DNA replication, and repairing DNA damage.	ARID1B	Ovarian cancer	^79^
			EZH2	Ovarian cancer	^148^
			PARP1	Breast and colon cancer	^149^
MAD2 (overexpress)	4q27	A component of the mitotic spindle assembly checkpoint that prevents the onset of anaphase until all chromosomes are properly aligned at the metaphase plate.	PP2A	Lung and liver cancer; malignant lymphoma	^73^
CKS1B (overexpress)	1q21	Codes for a conserved regulatory subunit of cyclin-CDK complexes that function at multiple stages of cell cycle progression	PLK1	Breast cancer	^41^
TDP1 (overexpress)	14q32.11	Encode the protein that repairs stalled topoisomerase I-DNA complexes and repair of free-radical mediated DNA double-strand breaks.	HDAC1/2	Fibrosarcoma; rhabdomyosarcoma	^74^
			RPD3		

SL between homologous recombination-related gene BRCA1/2 and PARP is a classical and fundamental example. Several research groups first reported in 2005 that dysfunctional BRCA1 or BRCA2 cells are significantly more sensitive to PARP inhibitors than cells that have normal BRCA function.^35,36^ PARP1 is a DNA repair protein that regulates cell proliferation and differentiation by repairing DNA single-strand break (SSB) and double-strand breaks (DSB). The inhibition of PARP1 leads to deleterious mutation accumulation, resulting in the apoptosis of BRCA1/BRCA2-deficient cells.^44^ Subsequently, the specific mechanisms of SL between these two genes were further investigated and will be discussed at the pathway level later. After PARP inhibitors demonstrated its feasibility, acceptable safety, and considerable efficacy,^45^ the development of PARP inhibitors increased rapidly and several drugs were evaluated for their use on a wide range of solid tumors and hematologic cancers in clinical trials (Table 2).^46–51^

**Table 2:** Recent clinical trials potentially related to synthetic lethal interactions

Gene	Targeted SL partners	Agent	Intervention	Cancer type	Phase and ClinicalTrials.gov Identifier
BRCA1/2	PARP	Olaparib	Olaparib	Breast and ovarian cancer	IV, NCT04330040
			Olaparib + Paclitaxel + Durvalumab	Advanced gastric cancer	II, NCT03579784
			Olaparib + Abiraterone	Prostate cancer	III, NCT03732820
			Olaparib + Durvalumab	Bladder cancer	II, NCT03534492
			Olaparib + Temozolomide	Colorectal cancer	II, NCT04166435
		Niraparib	Niraparib	Pancreatic cancer	II, NCT03601923
			Niraparib + Osimertinib	Lung cancer	I, NCT03891615
			Niraparib + Dostarlimab	Ovarian cancer	III, NCT03602859
			Niraparib + MGD013	Gastric and Gastroesophageal junction cancer	I, NCT04178460
			Niraparib + Dostarlimab	Cervix cancer	II, NCT04068753
		Rucaparib	Rucaparib	Endometrial cancer	II, NCT03617679
			Rucaparib + Nivolumab	Biliary cract cancer	II, NCT03639935
			Rucaparib + Radiotherapy	Breast cancer	I, NCT03542175
			Rucaparib + Copanlisib	Prostate cancer	I, NCT04253262
			Rucacparib + Enzalutamide + Abiraterone	Prostate cancer	I, NCT04179396
		Talazoparib	Talazoparib	Leukemia	I, NCT03974217
			Talazoparib + Avelumab	Breast cancer	I, NCT03964532
			Talazoparib + Radiotherapy	Gynecologic cancer	I, NCT03968406
			Talazoparib + ASTX727	Breast cancer	I, NCT04134884
			Talazoparib + Avelumab	Lung cancer	II, NCT04173507
			Talazoparib + Axitinib	Kidney cancer	I/II, NCT04337970
			Talazoparib + Atezolizumab	Lung cancer	II, NCT04334941
			Talazoparib + Gedatolisib	Breast cancer	II, NCT03911973
TP53	ATR	Berzosertib (M6620)	Berzosertib + Radiotherapy	Lung and breast cancer	I, NCT02589522/ I, NCT04052555
			Berzosertib + Topotecan (Hydrochloride)	Lung cancer	I/II, NCT02487095/ II, NCT03896503
			Berzosertib + Carboplatin + Docetaxel	Prostate cancer	II, NCT03517969
		AZD6738	AZD6738 + Radiotherapy	Advanced solid tumors	I, NCT02223923
			AZD6738+ Olaparib	Gynecologic cancer	II, NCT04065269
			AZD6738 + Olaparib + Durvalumab	Breast cancer	II, NCT03740893
			AZD6738 + Acalabrutinib	CLL	I/II, NCT03328273
			AZD6738+ Durvalumab	Biliary tract cancer	II, NCT04298008
		BAY1895344	BAY1895344	Advanced solid tumors	I, NCT03188965
			BAY1895344 + Pembrolizumab	Advanced solid tumors	I, NCT04095273
			BAY1895344 + Niraparib	Ovarian cancer	I, NCT04267939
		M4344	M4344 + Niraparib	Ovarian cancer	I, NCT04149145
			M4344 + Carboplatin	Advanced solid tumors	I NCT02278250
	WEE1	Adavosertib (AZD1775)	Adavosertib	Advanced solid tumors	I, NCT01748825; II, NCT03253679 / NCT03284385
			Adavosertib + Gemcitabine + Cisplatin + Carboplatin	Advanced solid tumors	I, NCT00648648
			Adavosertib + Olaparib	Ovarian, primary peritoneal, and fallopian tube cancer	II, NCT03579316
				Advanced solid tumors	II, NCT02576444
			Adavosertib + Olaparib + AZD6738	Breast cancer	II, NCT03330847
			Adavosertib + Irinotecan	Advanced solid tumors	I/II, NCT02095132
			Adavosertib + Cisplatin + Radiotherapy	Cervical, vaginal, and uterine cancer	I, NCT03345784
			Adavosertib + Temozolomide + Radiotherapy	Glioblastoma	I, NCT01849146
	CHK1	SRA737	SRA737	Advanced solid tumors	I/II, NCT02797964
			SRA737 + Gemcitabine + Cisplatin	Advanced solid tumors	I/II, NCT02797977
		Prexasertib (LY2606368)	Prexasertib	Advanced solid tumors	I, NCT01115790
				Lung cancer	II, NCT02735980
				Breast, ovarian, and prostate cancer	II, NCT02203513
	mTOR	Temsirolimus	Temsirolimus	Endometrial carcinoma	II, NCT02093598
		Metformin	Metformin + Carboplatin + Paclitaxel	Epithelial ovarian cancer	II, NCT02312661
KRAS	PLK1	CYC140	CYC140	Myelodysplastic syndromes, AML, ALL, CML, CLL	I, NCT03884829
		BI 2536	BI 2536	Pancreatic neoplasms	II, NCT00710710
		BI 6727	BI 6727	Neoplasms	I, NCT01145885
		NMS-1286937	NMS-1286937	Advanced or metastatic solid tumors	I, NCT01014429
		GSK461364	GSK461364	Non-Hodgkins lymphoma	I, NCT00536835
		Onvansertib (PCM-075)	Onvansertib + Cytarabine+ Decitabine	AML	I/II, NCT03303339
	CD274/PD-L1	Sotorasib (AMG 510)	Sotorasib + MEK inhibitor; Sotorasib + PD1 inhibitor; Sotorasib + SHP2 allosteric inhibitor; Sotorasib + Pan-ErbB tyrosine kinase inhibitor; Sotorasib + PD-L1 inhibitor; Sotorasib + EGFR inhibitor + Chemotherapy	Advanced solid tumors	I, NCT04185883
		Pembrolizumab	Pembrolizumab + Docetaxel + Ramucirumab	NSCLC	II, NCT04340882
			Pembrolizumab + Trametinib		I/II, NCT03225664; I, NCT03299088
		Durvalumab	Durvalumab + Carboplatin + Pemetrexed	Lung cancer	II, NCT04470674
		Avelumab	Avelumab + Binimetinib + Talazoparib	Pancreatic cancer	II, NCT03637491
MYC	4EBP1	AZD2014	AZD2014	Prostate cancer	I, NCT02064608
		CC-115	CC-115	Glioblastoma multiforme, squamous cell carcinoma of head and neck, prostate cancer, Ewing’s osteosarcoma, and CLL	I, NCT01353625
		Everolimus	Everolimus + Nelarabine + Cyclophosphamide + Etoposide	Lymphoblastic leukemia and lymphoblastic lymphoma	I, NCT03328104
	AURKB	GSK1070916A	GSK1070916A	Adult solid tumor	I, NCT01118611
	CDK9	AZD4573	AZD4573	Relapsed or refractory hematological malignancies and Richter’s syndrome	I, NCT03263637
		TP-1287	TP-1287	Advanced solid tumors	I, NCT03604783
		P276-00	P276-00	Melanoma	II, NCT00835419

The tumor-suppressor gene TP53, a predominant target in SL research, is the most frequently mutated gene in cancers.^52^ Identifying the synthetic lethal partners of p53 is a feasible method in clinical practice. A previous study conducted by Wang and Simon used gene-expression profiling to select multiple candidates for synthetically lethal gene targets of p53.^53^ A series of kinase-encoding genes were found to be potential targets of p53-deficient tumors for new drug therapy, including polo-like kinase 1 (PLK1), cyclin-dependent kinase 16 (CDK16), receptor-like tyrosine kinase (RYK), aurora kinase A (AURKA), etc. Recently, increasing studies reported new synthetic lethal partners of p53 such as ATM, ATR, WEE1, CHK1, etc.,^54–58^ in various types of cancers (listed in Table 1). Furthermore, Pan et al. revealed a different synthetic lethal therapy between p53 and B-cell lymphoma 2 (BCL-2) by activating p53 through MDM2 silencing and inhibiting BCL-2, which accelerates the apoptosis process in acute myeloid leukemia (AML) cells.^59^ This indicates that a combination of targeting mutated genes with their synthetic lethal partners may improve the synthetic lethal effects in more cancers than only inhibiting partner genes as in previous studies.

SL can also be applied to target oncogenesis drivers such as KRAS and MYC. Recent studies have identified synthetic lethal partners for oncogene KRAS, which was not considered “druggable” by traditional chemotherapy,^60^ by using large-scale RNAi screening. An earlier study suggested that serine-threonine kinase 33 (STK33) was indispensable for the viability of KRAS-driven tumors; however, this result is considered controversial according to a later study. Previous studies have proved this synthetic lethal effect in colon cancer (DLD-1, HCT-116, SW-480), pancreatic cancer (PANC-1), lung cancer (A549), and other cell lines.^61,62^ However, some of these cell lines were not verified in the later study. Therefore, the discrepancy of the cell lines and cancer types used in the two studies may be the cause for the opposite result, which suggests that more researches are needed to confirm the application of synthetic lethal effects in various cell lines of the same cancer and in different cancer types. Through RNAi assay, Steckel et al. conducted a series of studies to illuminate the synthetic lethal interactions that DNA replication regulator CDC6 and transcription factor GATA2 have with KRAS.^63,64^ Downstream regulatory pathways of GATA2 were further studied in NSCLC, which are described in the next section—synthetic lethal pathways. Moreover, SLC25A22 has been identified as a synthetic lethal gene in colorectal cancer cells with KRAS mutations.^65^ The inhibition of PLK1 and RhoA/Rho kinase (ROCK) has a synergistic effect in KRAS-mutant cancers,^66^ which is a more complex condition of SL in genetics involving these three genes. Recently, CD274 (encoding PD-L1) blockade has been proved to be a promising KRAS-mutant adenocarcinoma treatment option.^67^ MYC-targeted therapies, similar to KRAS-targeted therapies, have proven to be a challenge to explore. Recent studies suggest candidate genes that are synthetically lethal in MYC-driven cancers. These include eukaryotic translation initiation factor 4E (eIF4E) binding protein 1 (4EBP1), SUMO-activating enzyme subunit 1/2 (SAE1/2), Aurora-B kinase (AURKB), PIM1, and Cyclin-dependent kinase 9 (CDK9).^68–72^

Furthermore, mitotic arrest deficiency 2 (MAD2) shares synthetic lethal interaction with PP2A, in which PP2A inhibition in MAD2 overexpressing tumor cells results in SL in several tumors, including lung cancer, liver cancer, and malignant lymphoma.^73^ Likewise, PLK1 inhibition in CKS1B overexpressed tumor cells leads to breast cancer-cell death, and the inhibition of histone deacetylases (HDACs) or histone deacetylase RPD3 in TDP1 overexpressed cells may kill fibrosarcoma and rhabdomyosarcoma cells.^41,74^ Several studies referred to these interactions as SDL, an expanding concept of SL, in which the overexpression of one gene combined with the loss of function in another gene that results in cell death, and therefore could be used to target cancer cells with overexpressed, undruggable oncogenes.^75^ Intriguingly, SDL is without doubt subordinate to the basic concept of SL mentioned above (Fig. 1a) and can be classified into the category of SL in genetics.

## Molecular and cellular level in synthetic lethal pathway

Various pathways are crucial for survival in both normal and cancer cells. Proteins are synthesized as a product of multiple gene expression and form the basis for these functional pathways. Ku et al.^76^ found that the established synthetic lethal effects at the pathway level are more reproducible than those previously reported at the gene level through analysis. Consequently, after identifying genes that have synthetic lethal interactions in tumors, the mechanisms involved in these vital pathways, which these genes participate in, are then studied further by researchers. Pathway mechanisms are primarily studied at the protein level. This section will discuss the classification of the findings of SL at the pathway level.

### Single synthetic lethal pathway

**Fig. 2:**
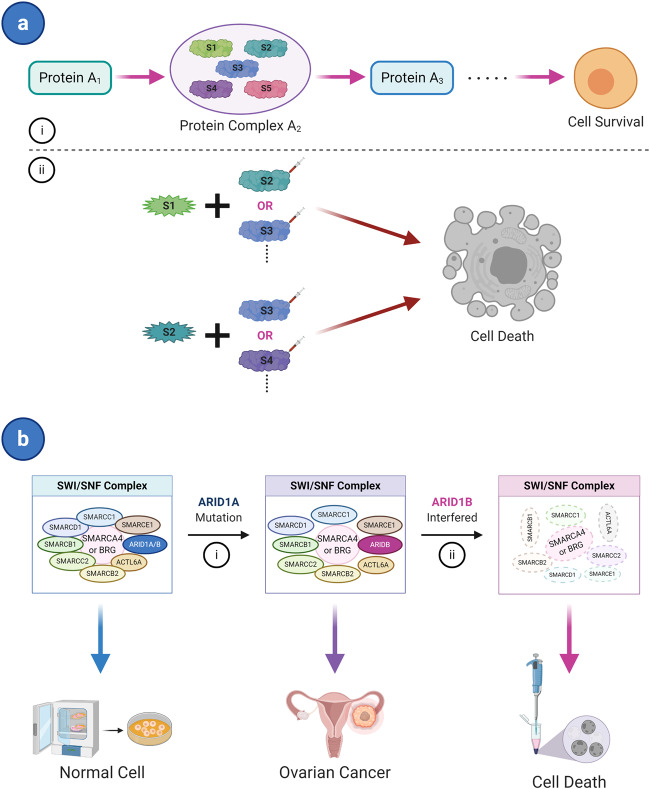
Synthetic lethal pathway: single pathway. **a** Single pathway concept. (i) A pathway performs an essential survival function to maintain cell survival; Protein complex A2 formed by the joint expression of multiple genes (S1, S2, S3, etc.) is an essential factor of this pathway. (ii) Abnormality (mutation, overexpression, or inhibited) of two or more genes in the complex leads the cell death, while only one mutated gene of the complex is viable. **b** Examples of the SWI/SNF complex. (i) Mutation of the ARID1A subunit of the SWI/SNF complex may turn normal cells into cancers like ovarian cancer and tumor cells still survive. (ii) Inhibition of ARID1B, another subunit of the SWI/SNF complex, will cause the complex collapse and synthetic lethality. Star shape of genes represents a mutation; syringe represents inhibitors; viable cells are depicted as ovals; and inviable cells are depicted as random shapes

Functionally related genes are translated into proteins in sequence to form a pathway that performs essential functions within a cell. In many cases, several components of these pathways are complexes formed by the collaborative expression of multiple genes. Abnormality in two or more genes that constitute the same protein complex on a pathway may lead to cell death. Fang^77^ also reported that the synthetic lethal effect in essential multiprotein complex subunits that is a component of a single linear essential pathway is a condition of SL. Therefore, this synthetic lethal effect mainly focuses on a pathway (Fig. 2a).

The switch/sucrose non-fermentable (SWI/SNF) chromatin remodeling complex is assembled by its subunit proteins that some genes, such as SMARCA2/4, SMARCB1, ARID1A/B, and ACTL6A, encode. SWI/SNF complex mainly participates in DNA replication and repair.^6,78,79^ The gene that encodes AT-rich interactive domain 1 A (ARID1A), a member of the SWI/SNF complex, is frequently mutated across a variety of human cancers. Helming et al. identified that ARID1B knockdown in ARID1A-mutant ovarian cells leads to dissociation of the core catalytic ATPase subunit SMARCA4 (or BRG1) and reduced combination of other subunits in the SWI/SNF complex (Fig. 2b). It was observed that the proliferation of tumors was inhibited.^79^ Based on this finding, SL between ARID1A and ARID1B could possibly be expanded and applied to more ARID1-mutant tumor cells such as hepatocellular carcinoma (HCC) and colon cancer.^80^ The other two subunits SMARCA2 and SMARCA4 that make up the SWI/SNF complex share a similar relationship, as SMARCA2 is essential for the survival of tumor cells that possess function mutations in SMARCA4.^81,82^

Another example of synthetic lethal effect via a pathway was recently reported in pancreatic ductal adenocarcinoma (PDAC). Somatic mutations in SMAD4 are often associated with PDAC.^83^ Dey et al. studied metabolic gene malic enzyme 2 (ME2) at the SMAD4 site and its paralogous isoform ME3 in PDAC. ME2 and ME3 are both oxidative decarboxylases that are expressed in the same metabolic pathways to catalyze the conversion of malic acid to pyruvate, in which the loss of ME3 causes ME2-mutated PDAC cell death.^43^

Interestingly, the examples mentioned above are paralogous genes and take part in collateral SL. In collateral SL, mutation or deletion of the gene that encodes a subunit in a specific complex often causes the collateral or passenger gene to become vulnerable. The subunit translated by this collateral gene is a component of the same complex. Further inhibition of the collateral gene causes this complex to collapse and results in the collateral SL of cells.^6,84^ Collateral SL is consistent with single pathway SL (Fig. 2), thus it can be grouped into this category of SL.

### Dual synthetic lethal pathway

**Fig. 3:**
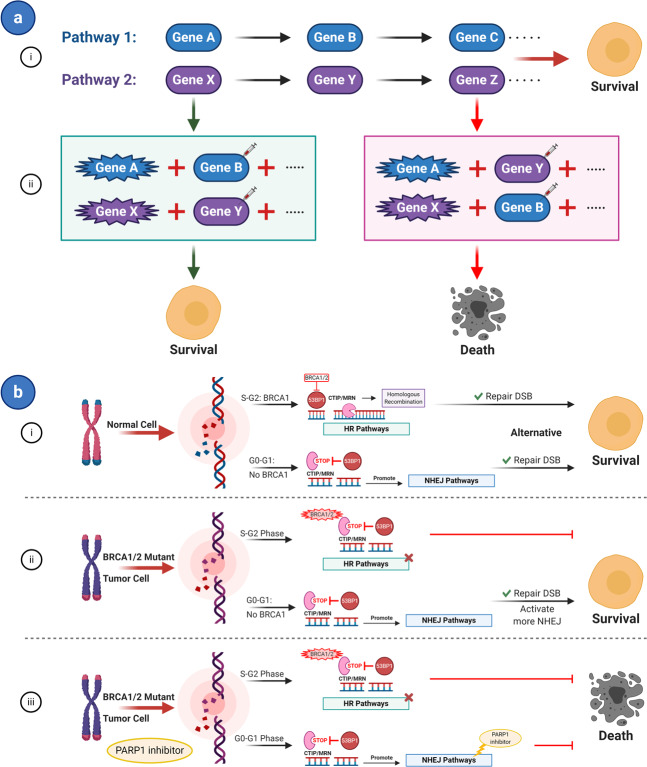
Synthetic lethal pathway: dual pathways. **a** Dual pathway concept. (i) Pathways 1 and 2 perform the same function to maintain cell survival. (ii) Abnormality (mutation, overexpression, or inhibited) of two or more genes in only one pathway keeps the cell viability. On the contrary, two or more genes on two pathways in abnormal conditions would cause synthetic lethal interactions. **b** Examples of HR and NHEJ pathways. (i) When DSBs occur in normal cells, BRCA1 is normally expressed and is recruited to sites of breaks, which interacts with 53BP1 to inhibit 53BP1 on the CTIP/MRN complex that promotes end processing to allow HR-mediated repair in S and G2 phases. Whereas in the G0/G1 phase, BRCA1 is silent and 53BP1 is recruited to DSBs to restrain CTIP/MRN activity, which inhibits HR and promotes the classic-NHEJ pathway. (ii) In BRCA-mutated tumors, BRCA1 is not present in S/G2-phase and 53BP1 inhibits CTIP/MRN function, leading to impaired end processing of the breaks, suppression of HR, and promotion of the alternative-NHEJ pathway. In this condition, tumors could still rely on the alternative-NHEJ pathway to repair DSBs and survive. (iii) Use of PARP (a functional gene in the NHEJ pathway) inhibitors will cause synthetic lethality in BRCA-mutated cancers. Star shape of genes represents a mutation; syringe represents inhibitors; viable cells are depicted as ovals; and inviable cells are depicted as random shapes

This type of SL involves two or more genes and two pathways. Specifically, two pathways perform the same survival function to maintain the cell alive, and abnormality of two or more genes that are key regulatory points in two pathways will cause synthetic lethal interactions in tumors, while the abnormality of genes in only one pathway maintains survival (Fig. 3a).

The synthetic lethal interactions between two major pathways of DNA DSBs repair, homologous recombination (HR) pathway and non-homologous end-joining (NHEJ) pathway,^85,86^ belong to this category. When DBSs occur in normal cells, BRCA1 is activated in response to DNA damage and recruited at breakpoints to inhibit 53BP1 on the end processing promoting complex C-terminal binding protein interacting protein (CTIP)/ Mre11-Rad50-Nbs1 (MRN), thus allowing HR-mediated repair during S and G2 phases of the cell cycle.^87,88^ In contrast, BRCA1 is silent in G0 and G1 phases, and 53BP1 is recruited to DSBs to inhibit CTIP/MRN activity, thus inhibiting HR and promoting classic (c)-NHEJ pathway (Fig. 3bi).^89–91^ In BRCA-mutated tumors, BRCA1 is not present in either S or G2-phases and 53BP1 remains free to inhibit CTIP/MRN function, leading to impaired end processing of breaks, suppression of HR, and promotion of alternative (Alt)-NHEJ pathway.^90,91^ In this scenario, tumors can rely on the (Alt)-NHEJ pathway to repair DSBs and survive (Fig. 3bii). However, PARP (a functional gene in the NHEJ pathway)^85^ inhibitors causes SL in BRCA-mutated cancers (Fig. 3biii).^36^ Further research of the HR pathway revealed that the microhomology-mediated end-joining (MMEJ) pathway also have synthetic lethal interactions with it. Ceccaldi et al. revealed that knockdown of DNA polymerase θ (Polθ also known as POLQ) belonging to MMEJ pathway in HR-deficient epithelial ovarian cancers enhances cell death.^92^

In addition, p53 and MAPKAP kinase-2 (MK2) also has a synthetic lethal effect between two pathways.^39^ CDK2 function is inhibited by p21, a downstream target of p53, which interacts with cyclin A and cyclin E to facilitate normal cell cycle entry through participation in the formation of the circ-Foxo3-p21-CDK2 ternary complex. This pathway is regulated by p53 that activates cell cycle checkpoints by inducing cell cycle arrest, thus providing time for DNA damage repair.^38,93,94^ In contrast, p53-deficient tumors are specifically dependent on the p38/MK2 pathway to prolong G2/M and G1/S checkpoints in response to DNA damage.^95^ Therefore, MK2 inhibition to block the p38/MK2 pathway could produce a synthetic lethal effect after DNA damage in p53-mutated NSCLC and glioblastoma cells.^96,97^ To sum up, the synthetic lethal interactions between two pathways that could regulate the same essential survival function of cells belong in this classification.

### Multiple synthetic lethal pathway

**Fig. 4:**
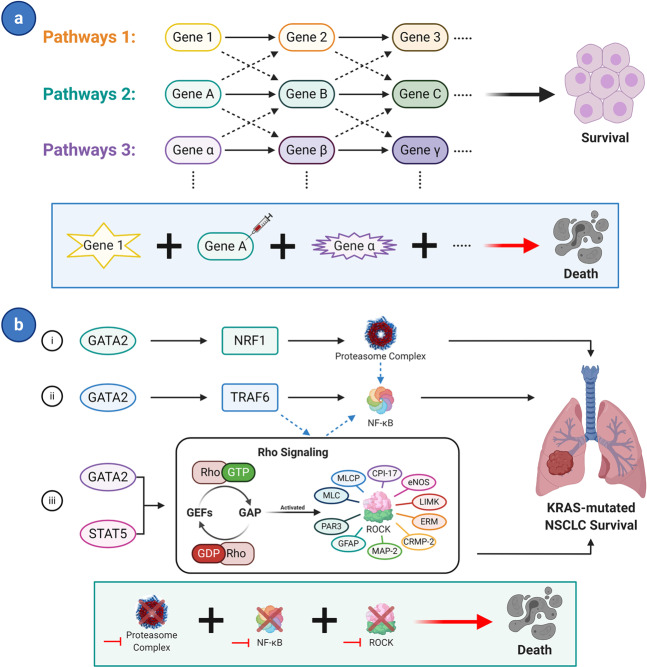
Synthetic lethal pathway: multiple pathways. **a** Concept of connected multiple pathways. Some pathways form a network and perform their functions to maintain cell survival. Whereas the presence of abnormal (mutation, overexpression, or inhibited) genes in every pathway leads to cell death. However, cells could still survive with abnormal genes in several but not all pathways. **b** Example: Survival of KRAS oncogene-driven NSCLC depends on the GATA2 transcriptional network. In KRAS-mutated NSCLC, three GATA2 downstream pathways (proteasome pathway, Rho-signaling cascade, and NF-κB signaling pathway) and related cross-talk are essential for the viability of tumors. Combined utilization of Bortezomib (inhibit proteasome and NF-κB) and Fasudil (inhibit Rho-signaling cascade) leads the tumors to death, whereas a single drug could not kill the tumors. Star shape of genes represents a mutation; syringe represents inhibitors; solid black arrows indicate directions of regulation; and dashed lines indicate cross-talk among pathways

Aside from SL involving just one or even two pathways as mentioned above, more intricate synthetic lethal interactions involving multiple pathways were identified through further study. Researchers have found that several tumors depend more on some pathways to survive than normal cells and considerable “cross-talk” exists among these pathways at the same time.^39^ The network of these pathways maintains vital functions in tumors. The co-suppression of these pathways leads to SL while blocking a single pathway or several but not all pathways do not.^64^ This type of SL involving multi-pathways is described in Fig. 4a.

One such example is the synthetic lethal effect of the GATA2 transcriptional network in KRAS-mutated NSCLC. Steckel et al. initially demonstrated that transcription factor GATA2 is requisite for KRAS oncogene-dependent cancer cells through RNAi assay.^63^ Because there is no clinical targeted drug for GATA2, the downstream regulatory pathways of GATA2 were studied. Proteasome pathway, Rho-signaling cascade, NF-κB (nuclear factor kappa light-chain enhancer of activated B cells) signaling pathway, and related cross-talk were proven by Downward and coworkers to be essential for KRAS-mutated NSCLC viability. Each independent member of this three-pathway network is not necessary for mutant NSCLC survival, thus inhibiting only one or two of the pathways does not lead to tumor death. However, the combined suppression of all three pathways will result in cell death (Fig. 4b).^64^ This synthetic lethal effect does not work on normal lung cells or non-KRAS-mutated NSCLC. Therefore, we assume that this type of SL could be a future direction for further research. Identifying more complex synthetic lethal networks may provide more targets for anticancer therapy.

## Organelles-targeted synthetic lethality

Recently, many researchers have explored SL targeting organelles,^98–102^ a more macro approach compared to synthetic lethal interactions in genes or functional pathways. This type of SL focuses on affecting or utilizing the major functions of organelles to cause tumor cell death. Currently, various experiments regarding SL are targeting mitochondrial function, which belongs to the category of “metabolic SL”, as referred to by some scientists.^98,103,104^ Herein, we will mainly describe specific examples of mitochondria-targeting SL, whose mutated metabolic enzymes cause cancers. In addition, other organelles-targeted SL will also be discussed.

**Fig. 5:**
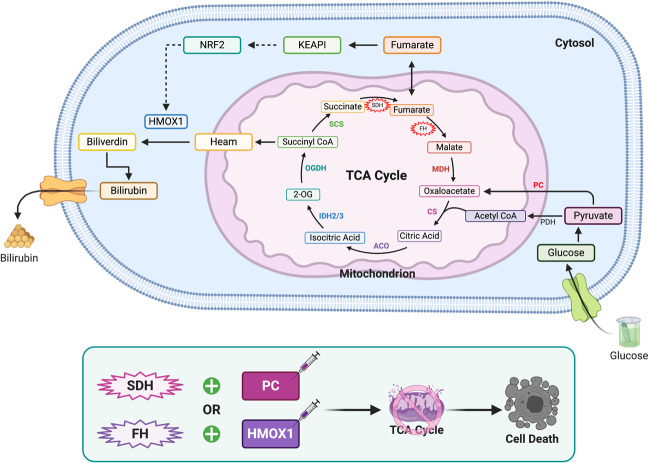
Organelles-targeted synthetic lethality—mitochondria. In SDH or FH mutant cancers, the main metabolic and signaling pathways involved in the metabolic reprogramming of SDH and FH related to mitochondria are presented above. Use of PC inhibitors in SDH-deficient tumor cells or inhibition of HMOX1 in FH-mutated cancers will disturb the TCA cycle, resulting in synthetic lethality. Solid black arrows indicate single step metabolic reactions; dashed black lines indicate indirect transcriptional cascades; star shape of genes represents a mutation; and syringe represents inhibitors. ACO (aconitase); CS (citrate synthase); FH (fumarate hydratase); HMOX1 (heme oxygenase 1); IDH (isocitrate dehydrogenase); KEAP1 (Kelch-like ECH-associated protein 1); MDH (malate dehydrogenase); NRF2 (nuclear factor erythroid 2-related factor); 2-OG (2-oxoglutarate); OGDH (oxoglutarate dehydrogenase); PC (pyruvate carboxylase); PDH (pyruvate dehydrogenase); SCS (succinyl-CoA synthetase); SDH (succinate dehydrogenase)

Succinate dehydrogenase (SDH), also known as mitochondrial respiratory complex II,^105^ is regarded as one of the most probable mitochondria-linked synthetic lethal targets. SDH is not only an essential mitochondrial enzyme in the tricarboxylic acid (TCA) cycle, it is also a key player in tumorigenesis. Previous studies demonstrated that SDH is inactive in SDH mutated tumors. This damages mitochondrial respiratory function through the shortening of the TCA cycle and abnormal accumulation of succinate.^105,106^ Although the metabolic adaptations that allow tumor cells to survive in SDH deficiency are not completely understood, recent studies have illustrated several important characteristics of SDH-deficient tumor cells. These experiments have demonstrated that SDHB-deficient tumor cells use more extracellular pyruvate than normal cells due to their insufficient biosynthetic capacity to meet the demands of this amino acid. These cells produce oxaloacetate, a fundamental factor in maintaining the aspartate level, and also transfer glucose-derived carbons for aspartate biosynthesis, which is critical for cell growth,^105^ through the preferential use of pyruvate carboxylase (PC).^106,107^ Furthermore, Cardaci et al. proved that PC inhibition not only reduced the proliferation of SDH-deficient tumor cells in vitro but also weakened the capability of these cells to form tumors in vivo.^106^ Therefore, PC shares a synthetic lethal interaction with SDH, whereby PC inhibition disturbs the TCA cycle (Fig. 5).

Additionally, mutations of mitochondrial enzymes such as fumarate hydratase (FH) and isocitrate dehydrogenases (IDH) are also involved in SL. FH is an enzyme of the TCA cycle that catalyzes the hydration of fumarate to malate.^108^ The inhibition of Heme Oxygenase 1 (HMOX1) results in a significant reduction in the growth of fumarate hydratase 1 deficient tumor cells but has little or no effect on normal cells.^99^ Thus, there is a synthetic lethal effect between HMOX1 and FH1 that targets mitochondrial function (Fig. 5). A study conducted by Chan et al. revealed that BCL-2 inhibitor ABT-199 combined with IDH mutations have considerable effects on AML treatment, it is yet another example of SL through mitochondrial metabolism.^100^ Except for the TCA cycle, glycolysis is another indispensable process and feasible target related to the mitochondria. For example, natural product englerin A (EA) was proved to activate protein kinase C-θ (PKCθ), which induces an insulin-resistant. Moreover, EA simultaneously activates the transcription factor heat shock factor 1 (HSF1), an inducer of glucose dependence. Therefore, through promoting glucose addiction and simultaneously limiting the tumor cells uptake of glucose, EA influences glycolysis and metabolism of mitochondria to have a synthetically lethal effect on highly glycolytic tumors.^109^

Aside from SL that targets mitochondrial functions, recent research concentrates on SL targeting other organelles. Zhao et al. found that the combination of SU11274 (MET inhibitor) and gefitinib (EGFR inhibitor) could synergistically influence the function of ribosomes to reduce the proliferation of triple-negative breast cancer (TNBC) by reducing the level of ribosomal protein S6 (RPS6).^101^ In addition, Cordani et al. demonstrated autophagy activation through the formation of autophagic vesicles, in which their fusion with lysosomes by mTOR inhibitor can repress p53- deficient lung, breast, and pancreas cancer-cell growth.^102^

## Conditional synthetic lethality

SL is known to be context-dependent. This context dependence refers to synthetic lethal partner genes of oncogenes and tumor-suppressor genes under the original concept of SL.^11^ However, in addition to the abnormities of synthetic lethal genes, the heterogeneity of tumor cells, its microenvironment, and external disturbances can affect genetic interactions, resulting in condition-dependent genetic interactions.^110,111^ Therefore, several synthetic lethal effects (at the gene, functional pathway, and organelle level) mentioned previously will be weaker or unachievable in the absence of particular conditions. This complex phenomenon was called context-specific or contextual SL in earlier studies,^6,39^ and conditional SL in recent studies.^7,112^ Conditional SL is a special synthetic lethal effect on tumor cells that also depends on internal or external circumstances (specific genetic backgrounds, hypoxia, high ROS, use of DNA-damaging agents, etc.) (Fig. 1b). Conditional SL could account for the variation in synthetic lethal effects observed in different tumor cells or different cell lines in the same cancer type. When resistance to synthetic lethal tumor-targeting drugs occurs, conditional SL could provide insight on how to solve this problem. In summary, conditional SL is one step further from nonconditional/original SL and will hold great prospects for treating tumors of various complex conditions in the future. We will provide specific examples of conditional SL in this section.

Different genetic properties can suppress synthetic lethal interactions, resulting in therapeutic resistance. As mentioned above, the utilization of PARP inhibitors could lead BRCA mutant tumor cells to SL by destroying the two main DSBs repair pathways, HR and NHEJ.^36,85^ However, the loss of 53BP1 can inhibit synthetic lethal therapy using PARP inhibitors on BRCA1/2-mutated breast cancer.^113–115^ In 53BP1^+/+^ cells, Bouwman et al. proved that the loss of 53BP1 in BRCA1/2-mutated cancers may lead to therapeutic resistance to PARP inhibitors or platinum agents.^115^ In addition, ATR, ATM, and RAD51 also correlate with PARP inhibitor resistance in BRCA1/2-mutated cancers.^116–119^ Although uncertainty remains in the underlying mechanism of HR pathway restoration in BRCA1/2-mutated cells with loss of 53BP1, ATR, ATM, or RAD51 after the use of PARP inhibitors, it is evident that these genes are indispensable internal conditions that the synthetic lethal effect of BRCA and PARP requires. Similarly, microsatellite instability (MSI), which results from deficient DNA mismatch repair (MMR), is also a special and essential genetic background for SL in several cancers. Recent studies revealed that targeting WRN helicase has a synthetic lethal effect on the viability of microsatellite instability-high (MSI-H) but not microsatellite stable (MSS) colorectal and endometrial cancer-cell lines.^120,121^

Other internal conditions also play important roles in conditional SL. A recent study found that acute and chronic hypoxia in the cellular microenvironment may decrease HR protein expression and its function, which sensitizes cells to PARP inhibition.^122^ This finding can be applied in the treatment of BRCA1/2-mutated tumor cells that are resistant to PARP inhibitors. Furthermore, the use of PARP inhibitors can be extended to tumor cells without BRCA mutations because most solid tumors contain hypoxic cells.^122^ Similarly, through further study of conditional SL, other internal conditions such as proteotoxic stress and metabolic stress may be proven to increase the range of synthetic lethal interactions.^123^

In addition to internal conditions, the effect of external factors on tumors, such as radiation and chemotherapy drugs, also have been studied. When exposed to DNA-damaging agents and ionizing radiation, the dependence of tumor cells on PARP, involved in the repair of DNA damage, was enhanced.^124^ Thus, tumor cells with mutant genes that are synthetic lethal partners of PARP will be more sensitive to PARP inhibitors under those specific conditions. For instance, Bailey et al. identified that cohesin component STAG2 has a synthetic lethal effect with PARP in glioblastoma. Meanwhile, STAG2-mutated glioblastoma cells are more easily destroyed by PARP inhibitors when using temozolomide, a DNA-damaging drug.^125^

## Synthetic lethality targeted drugs in clinical stages

With increasing preclinical studies in the field of SL, SL-targeted drugs in clinical practice have been developed. After Ashworth and Helleday^35,36^ demonstrated the synthetic lethal interactions of PARP inhibitors in BRCA1/2-deficient tumors in 2005, several inhibitors based on SL for targeted cancer therapy have been applied in clinical practice.

**Fig. 6:**
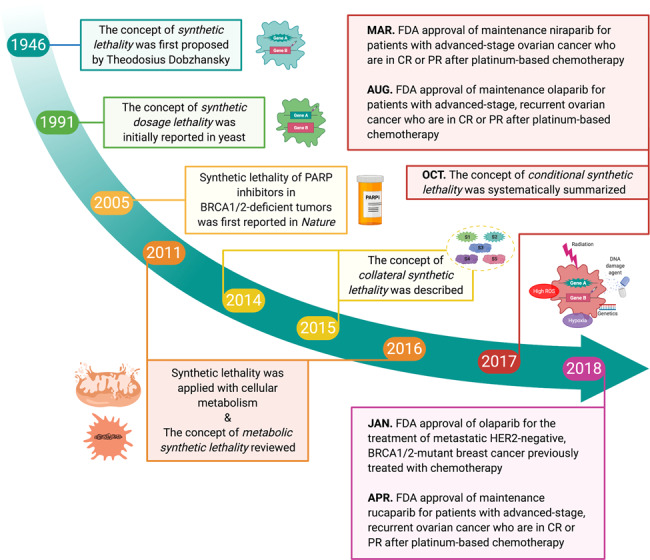
Timeline: landmark discoveries and advances of synthetic lethality in cancer. Several key events of synthetic lethality development. Several expanded concepts of SL beyond the original have been constantly proposed. In contrast, after Ashworth and Helleday demonstrated synthetic lethality of PARP inhibitors in BRCA1/2-deficient tumors, numerous studies on SL in cancer has been significantly increased and several inhibitors, especially PARPi based on SL, has been applied in clinical practice

Patients with both BRCA1 and BRCA2 mutations will usually suffer from a lifelong risk of breast and ovarian cancer.^126^ Initially, PARP inhibitors were applied as a combination therapy of low-dose rucaparib and full-dose temozolomide, a DNA alkylating agent, in clinical trials.^127^ In phase 1 olaparib clinical trial, which involved patients with BRCA1/2 mutations, 63% of the patients who received olaparib exhibited clinical benefit with minimal side effects than those of conventional chemotherapy regimens.^45^ Subsequently, phase 2 and 3 clinical trials, which included patients with BRCA1/2-mutated ovarian, breast, prostate and pancreatic cancers, demonstrated the clinical benefit offered by Olaparib.^128–132^ Based on these clinical trials, the FDA first approved olaparib for the treatment of advanced-stage, BRCA1/2-mutant ovarian cancers in 2014. Subsequently, olaparib was approved for patients with advanced-stage, recurrent ovarian cancer who are in CR or PR after platinum-based chemotherapy and metastatic HER2-negative, BRCA1/2-mutant breast cancer previously treated with chemotherapy in 2017 and 2018, respectively.^38^ The clinical trials and progress of FDA approval of other PARP inhibitors are presented in Table 2 and Fig. 6. In addition to PARP inhibitors, drugs targeting potential synthetic lethal partners of oncogenes or tumor-suppressor genes such as TP53, KRAS, MYC also have been tested in clinical practice (Table 2).

## Conclusion and future perspectives

Since the concept of SL was first proposed, the number of studies on this topic has significantly increased, and many expanded concepts of SL have been proposed. Furthermore, many synthetic lethal drugs, especially several PARP inhibitors approved by the FDA, have achieved major clinical breakthroughs (Fig. 6). In this review, we propose a new and systematic classification of SL that includes those expanded concepts of SL, such as synthetic dosage lethality, collateral SL, metabolic SL, etc. According to the specificity of research on its mechanism, various cases of SL were successively divided into gene-level SL, pathway-level SL, organelle-level SL, and conditional SL—from a more superficial to a deeper level. The classification we proposed above, along with the many recent findings of SL reviewed of each type, provide a more comprehensive understanding and many implications for the future perspectives on SL studies.

Firstly, the identification of SL interactions between genes is the primary step in SL at the gene level. It is imperative to identify more synthetic lethal effects among different genes in multiple cancers to develop a synthetic lethal gene database. In addition, verifying gene synthetic lethal effects in various cell lines of the same cancer and in different cancer types are also important. It could explain the controversies in previous studies, such as the synthetic lethal effect between STK33 and KRAS mentioned above, and provide the basis for further study on more complex conditions using the theory of conditional SL.^61,62^ Synthetic lethal screening technologies including drug screens,^133^ RNAi screens,^33^ bioinformatics screens,^34^ CRISPR screens,^32^ and combination of these methods,^11^ provide the possibility for geneticists to achieve this. At the gene level, many previous studies have focused on identifying a synthetic lethal relationship between two genes. The example of synthetic lethal effects among three genes (PLK1, ROCK, and KRAS) as mentioned above^66^ indicates that synthetic lethal interactions among multiple genes can be developed in the future. Similarly, previous studies also concentrated on searching for synthetic lethal partners of oncogenes while recent studies tend to target tumor-suppressor genes like p53. Identifying synthetic lethal partners of tumor-suppressor genes may have more potential to be explored. Besides, although targeting the commonly mutated and “undruggable” oncogenes and tumor-suppressor genes are invalid, from the example of activating p53 and inhibiting BCL-2 to kill AML,^59^ targeting these genes in combination with their synthetic lethal partners may significantly amplify the lethal effects on tumors compared to only inhibiting partner genes as in previous studies, which could expand the application of synthetic lethal effects in targeted cancer therapy.

Secondly, for researchers and clinicians working on targeted therapies for tumors, SL by pathway also provides many future perspectives. After identifying synthetic lethal partner genes, at protein or pathway level, they can further study those that are expressed unusually in specific cancer, while referring to the three synthetic lethal pathway conditions. Since SL has not been applied to the majority of cancers, the mechanism of the synthetic lethal pathway reported could be applied to less studied cancers that have the same mutated oncogenes or tumor-suppressor genes, such as nerve cancers, skin cancers, bone tumors, and biliary tract cancers. Furthermore, synthetic lethal effect in multiple pathways is an important direction in the future. The identification of difficult synthetic lethal effects can lead to further studies regarding pathway networks related to those difficult targets. As previously mentioned, Downward et al. revealed that the GATA2 downstream network (three pathways) has the same effect as GATA2 on KRAS-mutated NSCLC survival.^64^ Thus, there is a higher chance of finding points of the target for clinical inhibitors in each pathway. Furthermore, inhibitors approved by the FDA were used to block the three-pathway network, consequently killing KRAS-mutated NSCLC efficiently. Through this, the research achievements of SL could be better applied in clinical practice. In addition, organelle-targeted SL, the synthetic lethal effect that destroys mitochondria, ribosomes, lysosomes, and other organelles, is also a more macro direction of future research.

Lastly, conditional SL can provide explanations and future directions for the limitations of SL in anticancer therapeutic targets. The biggest challenge SL faces in clinical practice are drug resistance.^11,88^ In addition, synthetic lethal interactions that work in one cancer are sometimes ineffective in another. According to conditional SL, synthetic lethal effects on cancers require specific internal and external settings, which helps to explain these problems. Thus, exploring the specific circumstances required by the same cancers in different conditions or different cancer types is of great importance to solve drug resistance and expand the application of SL, which may be addressable using a multi-faceted testing framework.^76^

For preclinical studies, identifying different microenvironments and genetic backgrounds of cancer cells, which have different sensitivities to the same synthetic lethal effect, may reveal more drug resistance mechanisms.^134^ For clinical practice, a combination of external conditions including traditional chemotherapy drugs, immunotherapy, or radiation therapy coupled with SL-based drugs, holds great prospects to solve the issue regarding the resistance to synthetic lethal effect.^11,67^ In addition, due to the fact that high ROS is regarded as an internal condition to promote SL^7^ and several types of nanoparticles could generate ROS,^135^ the combination of synthetic lethal cancer therapy with nanotechnology could reduce drug resistance.^136^ However, Hocsak et al. demonstrated that PARP inhibitors suppress mitochondrial ROS production and decrease ROS-induced apoptosis in oxidative stress, thereby protecting the mitochondrial membrane potential via MKP-1 and ATF4 dependent pathway in A-549, T24/83, and WRL-68 human cell lines.^137^ Therefore, the role of ROS in conditional SL requires further studying and discussion. In summary, a great deal is yet to be understood and significant amounts of research to be done for tumor-targeted therapy still remains.

In conclusion, although SL is merely a simple genetic concept, its impact on cancer research has been increasing. This novel classification of SL along with the multiple findings of SL reviewed in each type mentioned above not only provides a systematic understanding of this field but also gives research more basis for reference and inspiration for future directions. We firmly believe that SL deserves further study and application in the field of cancer therapy in the future.
